# Tracking Signals of Change in Mediterranean Fish Diversity Based on Local Ecological Knowledge

**DOI:** 10.1371/journal.pone.0024885

**Published:** 2011-09-22

**Authors:** Ernesto Azzurro, Paula Moschella, Francesc Maynou

**Affiliations:** 1 Institut de Ciències del Mar (ICM-CSIC), Barcelona, Spain; 2 High Institute for Environmental Protection and Research (ISPRA), Laboratory of Milazzo, Milazzo, Italy; 3 Commission Internationale pour l'Exploration Scientifique de la Mer Méditerranée (CIESM), Monaco, Monaco; University of California, Berkeley, United States of America

## Abstract

One of the expected effects of global change is increased variability in the abundance and distribution of living organisms, but information at the appropriate temporal and geographical scales is often lacking to observe these patterns. Here we use local knowledge as an alternative information source to study some emerging changes in Mediterranean fish diversity. A pilot study of thirty-two fishermen was conducted in 2009 from four Mediterranean locations along a south-north gradient. Semi-quantitative survey information on changes in species abundance was recorded by year and suggests that 59 fish species belonging to 35 families have experienced changes in their abundance. We distinguished species that increased from species that decreased or fluctuated. Multivariate analysis revealed significant differences between these three groups of species, as well as significant variation between the study locations. A trend for thermophilic taxa to increase was recorded at all the study locations. The Carangidae and the Sphyraenidae families typically were found to increase over time, while Scombridae and Clupeidae were generally identified as decreasing and Fistularidae and Scaridae appeared to fluctuate in abundance. Our initial findings strongly suggest the northward expansion of termophilic species whose occurrence in the northern Mediterranean has only been noted previously by occasional records in the scientific literature.

## Introduction

Global change is having an ever increasing influence on the abundance and distribution of living organisms worldwide [1] but documenting the resulting biological trends is often constrained by the lack of information from studies at the appropriate temporal and spatial scales. The Increasing success of thermophilic biota colonizing the Mediterranean Sea [2] is one clear example of rapid changes that are happening at the regional scale. This is particularly evident when we look at fish colonization. Indeed a number of native species with tropical or subtropical affinity seem to have already moved towards the northern and colder sectors of the Mediterranean [3], [4]. This phenomenon, that has been named as “meridionalization” within the Mediterranean literature [5] is actually parallel to a number of poleward expansions of low latitude species that have been recorded all over the world [6], [7] for a variety of species such as plants [8], butterflies [9], birds [10], insects [11] and fish [12]. We are also expecting some cold water species to decline in the near future following forecast temperature rise [13]. Changes in the distribution of Mediterranean fishes are generally revealed by casual observation of scattered individuals outside the species known geographical range [14], being these sporadic records the only source of information that has been used to study meridionalization so far [4]. Consequently, the extent of these changes may be under appreciated, because of the limited and non-continuous nature of scientific monitoring [14]. The efforts that would be needed to monitor and survey marine habitats at scale large enough to perceive temporal and spatial trends is huge and this is clearly one of the major obstacles to researching global change [15]. There is an urgent need to fill this information gap by the use of new methodologies, suitable to deepen our capability to perceive the complex process of change.

In the last decades, “Local Ecological Knowledge” has emerged as an alternative information source on species presence or qualitative and quantitative indices of species abundance [16]–[22]. can be defined as the information that a group of people have about local ecosystems. We usually rely on knowledge gained by individuals over their lifetimes, and not what information has been handed down through the generations [23]. To extract data and information from individuals' memory, semi-structured or unstructured conversations between the researcher and a participant are commonly used, a practice commonly called “oral history” [24].

Here we aimed to explore of the utility of Local Ecological Knowledge to provide reliable information about some emerging changes in Mediterranean fish diversity. In the past, other studies used this approach to reconstruct trends in abundance of marine fish species, especially declines in abundance [25], [26], [27]. Our specific aims were threefold:

1. Identify indicators of meridionalization.
2. Reconstruct historical trends of abundance for indicator species.
3. Evaluate the potentiality of using Local Ecological Knowledge to the study large scale changes in Mediterranean fish diversity.

## Methods

### Study area

**Figure 1:**
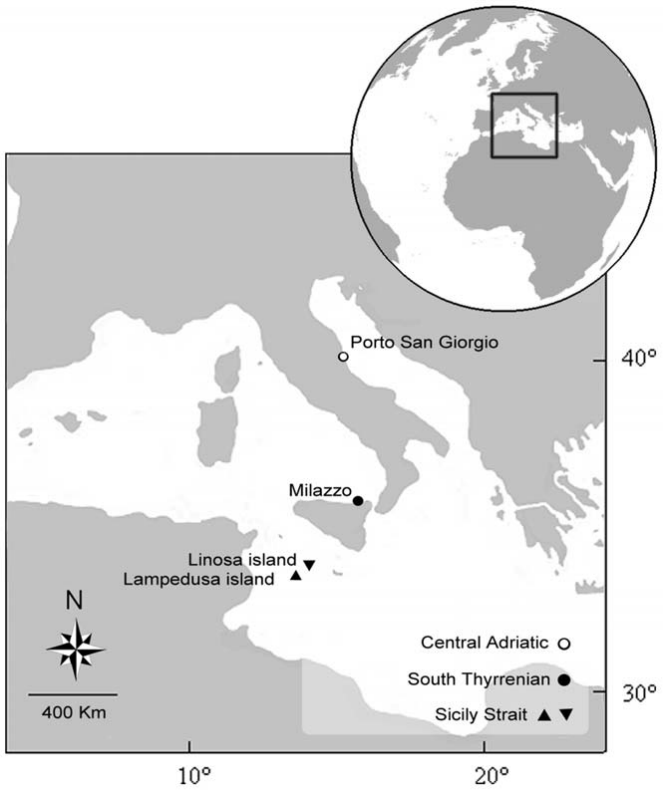
Study locations in the Mediterranean Sea.

Interviews were carried out between August 2009 and October 2010, over 4 different locations in the Mediterranean Sea (Figure 1): Linosa and Lampedusa, belonging to the archipelago of Pelagie Islands (Sicily Strait); Milazzo (Southern Tyrrhenian Sea) and Porto San Giorgio (Central Adriatic Sea).

### Sampling population

The study was directed to local and recreational fishermen with more than ten years of experience at sea. Fishermen were met during their land activities, while for example cleaning the nets in the harbors or fixing their boats. Special attention was placed to the approach, since fishermen generally mistrust fisheries researchers and managers. To counter this, formalities were avoided and conversations was focused on which species may have appeared or disappeared in the last decades. Once they showed interest in the subject through discussion then the interview was formally started. In other occasions, when we knew one local fisherman we asked him to introduce us to other fishermen.

### The survey questionnaire

People were interviewed on the basis of a detailed-protocol procedure (the “interview protocol” that has been officially adopted as part of the international basin-wide monitoring program “CIESM Tropical Signals” http://www.ciesm.org/marine/programs) and a standard questionnaire was developed around the following central questions:

“*Can you tell us what kind of fishes showed the greatest variation in abundance in the last decades*?” *Do you know species that have appeared or disappeared?*

The interview-protocol was developed to guide the interviewers in their task i.e. to extract the required information from the fisherman knowledge. At first, the fisherman's age and fishing gear used by the fisherman was recorded. Photographs from a field guide [28] were used to match local fish names with taxonomic ones. Only those species that were mentioned by the fisherman were registered. Respondents were also asked to provide a qualitative ranking of the abundance of these taxa through time, on an annual basis, according to 6 different grades: 0 = ABSENT; 1 = RARE (once in a year); 2 = OCCASIONAL (sometimes in a year); 3 = COMMON (regularly in a year); 4 = ABUNDANT (regularly in captures and abundant); 5 = DOMINANT (always in captures and with great abundances). At the end of each interview, each recorded taxa was assigned to a trend factor: species that “INCREASE” (level “I”); species that “DECREASE” (level “D”); and species that “FLUCTUATE (level “F”) over the respondent experience period.

### Conceptual approach for experimental design and statistical analysis

Considering the subjectivity of fishermen's knowledge we used a simple design in which the variability between the different interviews was taken into account and tested against our hypotheses (i.e. H_0_ of no differences between species groups; H_0_ of no differences between the different locations). The species mentioned in each interview were used to build a presence-absence dataset in which the “interviews” were the samples and the “species” the variables. Each interview taken from the same location was considered as an independent replicate sample. Finally our dataset was explored by means of multivariate and univariate analysis. A two way PERMANOVA [29], [30] based on Bray-Curtis resemblance matrix was used to test for the terms “Location” (with 4 levels) and “Trend” (with 2 levels: “Increase” or “decrease”) that were considered as fixed crossed factors. Fluctuating species were not included in the analysis because of the high number of empty cells. The Similarity Percentages Procedure (SIMPER) was used to identify the most important fish taxa in typifying the groups “I”, “D” and “F”. Cut off for low contributions was set at 90%. A Non-metric Multi Dimensional Scaling (nMDS) ordination was performed separately for the group of species “I” and “D” to visualize geographical patterns. All the multivariate analyses were performed with PRIMER 6+PERMANOVA software package from Plymouth Marine Laboratory, UK.

We applied breakpoint structural analysis [31], [32] to the time series of semi-quantitative abundance data to assess the year(s) of statistically significant change in abundance. For each year in the period 1969–2010 the median value of the semiquantitative abundance index was computed for species contributing to the typification of groups “I” and “D”. The technique of breakpoint analysis allows to identify statistically significant changes in the level of subsets of a time series. A time series is randomly split in 2 or more subsets (“data windows”) and the mean level compared through a modified F test (“structural change” or *sc test* [32]). The procedure is repeated iteratively until all significant breakpoints (if any) are identified [31]. In our case, we applied the Bayesian Information Criterion (BIC) as objective criterion to determine the number of breakpoints and their associated dates [32] with the corresponding 95% confidence intervals (CI). The breakpoint analyses were performed with the R library *strucchange*, developed by A. Zeileis at the University of Economics, Vienna (Wirtschaftsuniversität Wien, Austria).

## Results

**Figure 2:**
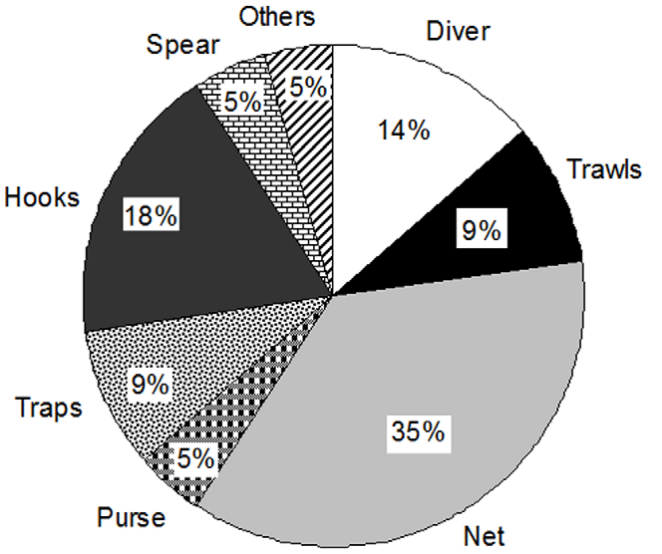
Percent distribution of fishing methods adopted by the respondents. Interviewed (Tot N = 32) were both recreational (N = 8, 25%) and professional (N = 24, 75%) fishermen.

A total of 32 artisanal fishermen were interviewed (9 from Linosa, 2 from Lampedusa, 10 from Milazzo and 11 from Porto San Giorgio). They were recreational (N = 8) and professional (N = 24) fishers with more than 10 years of experience at sea (15% N = 5 began before 1970; 28% N = 9 between 1970 and 1979; 38% N = 12 between 1980 and 1989; 19% N = 6 between 1990 and 1999) and an average age of 55 years. More than one fishing method was often adopted by the respondents, being the “nets” the most common fishing gear (35%) and diving performed by some of them (14%) (Figure 2). Out of 35 fishermen that have been contacted, only 3 refused to be interviewed. The duration of interviews ranged between 35 minutes and 1 h with an average of 44 minutes. Nevertheless, the whole amount of time needed to collect data included also the search for fishermen and informal conversations before and after the interview. On average we performed 4 interviews/day.

According to our compilation of local knowledge, 59 fish species belonging to 35 families resulted to have passed through drastic changes in their abundance (Table 1). Of them, 26 species fell in the group “INCREASE” (“I”); 42 in the group “DECREASE” (“D”) and 8 were indicated as “FLUCTUATE” (“F”). The total number of citations for these groups was: 91, 123 and 20 respectively.

**Table 1:** List of fish taxa cited by the respondents.

Taxa	I	D	F	Taxa	I	D	F
Ammodytidae		*5*		Pomatomidae	*6*		
*Gymnammodytes cicerelus*		5		*Pomatomus saltatrix*	6		
Atherinidae		*1*		Rajidae	*1*	*1*	
*Atherina sp*		1		*Raja sp*	1	1	
Balistidae	*6*			Scaridae	*8*		*5*
*Balistes capriscus*	6			*Sparisoma cretense*	8		5
Belonidae		*2*		Sciaenidae	*1*	*2*	
*Belone belone*		2		*Sciaena umbra*		2	
Carangidae	*24*	*5*		*Umbrina cirrosa*	1		
*Caranx crysos*	12			Scomberesocidae		*6*	
*Lichia amia*	4			*Scomberesox saurus saurus*	6		
*Seriola dumerilii*	4	3		Scombridae	*7*	*24*	*1*
*Trachinotus ovatus*	3			*Auxis rochei*		3	1
*Trachurus mediterraneus*	1	1		*Sarda sarda*	1	2	
*Trachurus trachurus*			1	*Scomber japonicus*		6	
Carcharhinidae		*1*		*Scomber scombrus*		17	
*Prionace glauca*		1		*Thunnus thynnus*		2	
Centracanthidae		*2*		Scophthalmidae	*1*		
*Spicara sp.*		2		*Scophthalmus rhombus*	1		
Clupeidae	*3*	*21*		Scorpaenidae		*1*	
*Engraulis encrasicolus*		12		*Scorpaena scrofa*		1	
*Sardina pilchardus*		4		Serranidae	*2*	*3*	*3*
*Sardinella aurita*	3			*E. marginatus*	2	3	3
*Sprattus sprattus*		5		Siganidae	*1*		*1*
Coryphaenidae	*6*			*Siganus luridus*	1		1
*Coriphaena hippurus*	6			Soleidae		*4*	
Dasyatidae	*2*	*6*		*Solea vulgaris*		4	
*Dasyatis pastinaca*	2	2		Sparidae	*7*	*16*	*1*
Exocoetidae		*4*		*Boops boops*	1	2	
Fistularidae			*7*	*Dentex dentex*		2	
*Fistularia commersonii*			7	*Diplodus annularis*		1	
Gadidae		*4*		*Diplodus sargus*	2	1	
*Merlangus merlangus*		2		*Diplodus vulgaris*	2		
*Trisopterus minutus capelanus*		2		*Lithognathus mormyrus*		2	
Gobiidae		*9*		*Oblada melanura*		2	
*Aphia minuta*		2		*Pagrus pagrus*		2	
*Gobius niger*		4		*Sarpa salpa*	1	4	1
*Gobius sp*		3		*Spondyliosoma chantarus*	1		
Labridae		*2*		Sphyraenidae	*14*		
*Coris julis*		2		*Sphyraena spp.*	14		
Merlucciidae		*3*		Syngnathidae		*3*	*1*
*Merluccius merluccius*		3		*Hippocampus sp*		2	1
Mugilidae	*3*			*Syngnathus sp*		1	
Muraenidae	*1*	*4*		Torpedinidae		*1*	
*Muraena helena*	1	4		*Torpedo sp.*		1	
Phycidae		*1*		Triglidae	*1*		*1*
*Phycis phycis*		1		*Trigla lucerna*	1		1

The number of times in which they were assigned to the groups ‘INCREASE’ (‘I’), ‘DECREASE’ (‘D’) and ‘FLUCTUATE’ (‘F’) is reported.

The Carangidae family had the most of citations for the group “I” (24 citations; 6 species) followed by Sphyraenidae (14 citations; 1 species). As for the group “D”, the mostly cited family was Scombridae (24 citations; 4 species) followed by Clupeidae (21 citations; 3 species), finally two families had the most of citations for the group “F”: Fistularidae (7 citations; 1 species) and Scaridae (5 citations; 1 species).

PERMANOVA analysis (Table 2) revealed significant differences for the terms “Location” and “Trend” and a significant interaction between these two factors.

**Table 2:** PERMANOVA Analysis.

Source	df	SS	MS	Pseudo-F	P(perm)
Location	3	26.582	8.8607	3.9332	0.001
Trend	1	10.378	10.378	4.6067	0.001
Location×Trend	3	25.388	8.4625	3.7565	0.001
Res	59	132.91	2.2528		
Total	66	203.82			

Permutational multivariate analysis of variance based on the Euclidean dissimilarity measure for presence-absence data. The test was done using 9999 permutations under the reduced model. The group ‘FLUCTUATE’ was excluded from the analysis.

**Figure 3:**
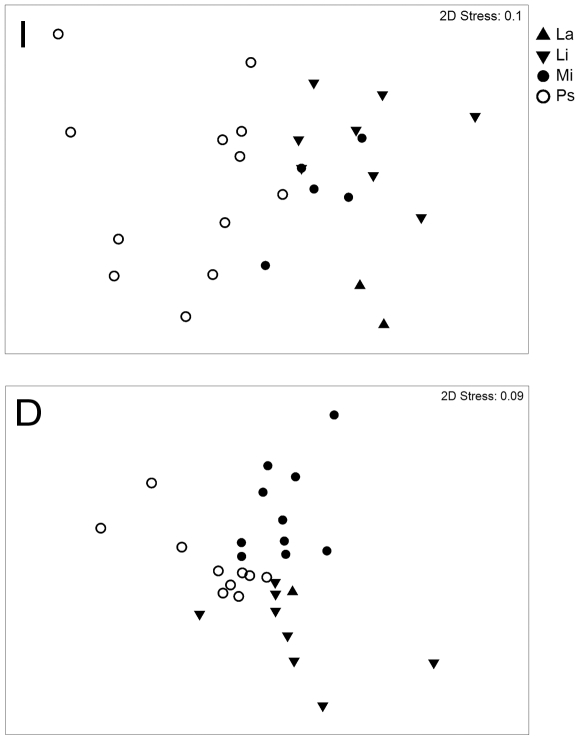
Non-metric Multi Dimensional Scaling (nMDS) ordination comparing interviews outputs across the different study locations. The position of each dot is defined by the assemblage of species recorded in each interview. **La** = Lampedusa; **Li** = Linosa; **Mi** = Milazzo; **Ps** = Porto San Giorgio. **I** = Group ‘INCREASE’; **D** = Group ‘DECREASE’.

The Non-metric Multi Dimensional Scaling (nMDS) ordinations showed the geographical structure of the “I” and “D” datasets (Figure 3) with no real prospect of a misleading interpretation. Looking at the “I” plot, it is quite clear the separation of the Adriatic location (Ps) from the remaining ones (Mi; La; Li) whilst in the “D” plot a separation between South Tyrrhenian (Mi) and the Pelagie islands (La and Li) seems to be apparent. According to SIMPER analysis (Table 3), 6 species contributed mostly to typify the group “I”; 11 species were important to characterize the group “D” and 3 species characterized “F”. The ‘I vs D’ average dissimilarity was of 98.85, highlighting the almost complete separation of “I” and “D” groups of species. Among the group “I”, two new fish species appeared (i.e. the species was cited from a location but was previously unknown by all the respondents) at Pelagie Islands (*Siganus luridus* and *Fistularia commersonii*). Four (*Caranx crysos*, *F. commersonii*, *Sparisoma cretense*. *Trachinotus ovatus*) were new to Milazzo and seven were new to Porto San Giorgio (*Balistes carolinensis*, *Coryphaena hippurus*, *Epinephelus marginatus*, *Lichia amia*, *Pomatomus saltatrix*, *Seriola dumerilii*, *Sphyraena viridensis*). No species were said to have completely disappeared or become locally extinct.

**Table 3:** Most important fish taxa in typifying the groups ‘I’ and D by SIMPER analysis.

Taxa	Av. frequency of occurrence	Contribution (%)	Group
*Sphyraena viridensis*	0.42	34.92	I
*Caranx crysos*	0.36	34.82	
*Sparisoma cretense* *	0.24	8.75	
*Coriphaena hippurus*	0.18	4.67	
*Balistes capriscus*	0.18	3.79	
*Scomber scombrus*	0.5	51.95	D
*Engraulis encrasicolus*	0.35	18.04	
*Scomberesox saurus saurus*	0.18	4.57	
*Gymnammodytes cicerelus*	0.15	3.07	
*Solea vulgaris*	0.12	2.45	
*Sarpa salpa*	0.12	2.45	
*Sprattus sprattus*	0.15	2.2	
*Muraena helena*	0.12	1.8	
*Gobius niger*	0.12	1.51	
*Sardina pilchardus*	0.12	1.51	
*Fistularia commersonii*	0.5	24.14	F
*Sparisoma cretense* §	0.36	22.17	
*Epinephelus marginatus*	0.21	16.26	

List of fish taxa in decreasing order of their importance in typifying the groups ‘INCREASE’ (‘I’) and ‘DECREASE’ (‘D’) by SIMPER analysis performed on presence/absence data. Cut off for low contributions: 90.00%. Group ‘I’ average similarity 18.05; Group ‘D’ average similarity 12.51; Group ‘F’ average similarity 15.44.

*Milazzo,

§ Linosa and Lampedusa.

**Figure 4:**
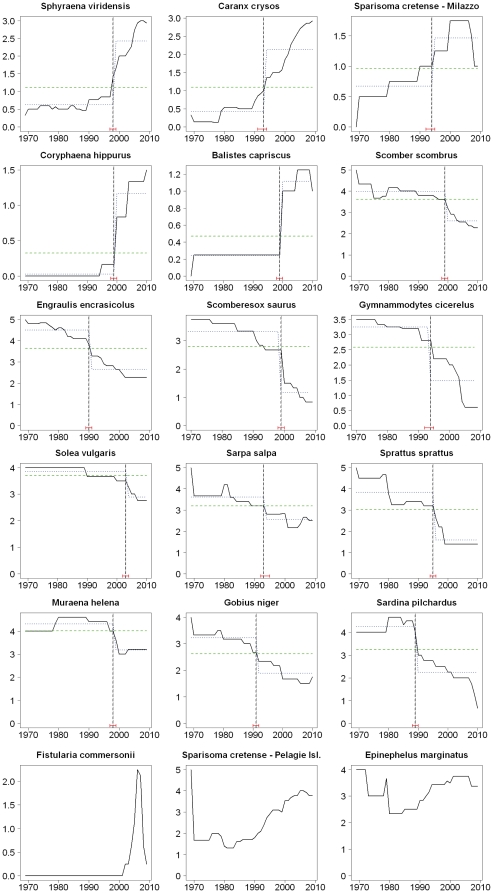
Dynamic of the abundance of ‘SIMPER species’, according to fisher's perceptions. Trends of relative abundance on a scale from 0 to 5 (see text) of the species contributing mostly to the SIMPER analysis. Bold continuous line: mean relative abundance; dashed green line: null model of no temporal change in relative abundance; dashed blue lines: best fitting local regressions before and after break point; vertical dashed line: breakpoint or year of significant change in the temporal evolution of abundance, with 95% confidence intervals in red brackets.

The time series of median abundance of the species most responsible for change, as identified in the SIMPER analysis, are shown in Figure 4, with the results of the breakpoint analysis given in Table 4. The analysis of the semiquantitative time series shows that most species undergoing an increase in abundance (and fluctuating species with recent increase), as well as many decreasing species, had significant breakpoints in the late 1990s. This figure also shows that a significant decrease in abundance happened in the 1990s for most commercial species: *Engraulis encrasicolus* from dominant to common in 1993, *Scomber scombrus* from abundant to occasional in 1999, *Solea vulgaris* from abundant to common in 1999, and *Sprattus sprattus* from dominant to rare in 1995; while some pelagic species increased in abundance slightly later: *C. hippurus* from absent to rare or occasional in 2003.

**Table 4:** Results of the breakpoint structural analysis.

Species	Trend	Year	Sup Ftest
*Balistes capriscus*	F+	1999 (1998–2000)	1060.94
*Caranx crysos*	I	1993 (1991–1994)	150.77
*Coryphaena hippurus*	I	1999 (1998–2000)	503.85
*Engraulis encrasicolus*	D	1990 (1989–1991)	257.69
*Gobius niger*	D	1991 (1990–1992)	195.78
*Gymnammodytes cicerelus*	D	1992 (1994–1995)	125.30
*Muraena helena*	D	1998 (1997–1999)	170.02
*Sardina pilchardus*	D	1989 (1988–1990)	201.95
*Sarpa salpa*	D	1993 (1992–1995)	86.03
*Scomber scombrus*	D	1999 (1998–2000)	145.62
*Scomberesox saurus*	D	1999 (1998–2000)	227.22
*Solea vulgaris*	D	2003 (2002–2004)	138.97
*Sparisoma cretense* (Pelagie Isl.)	F+	2001 (2000–2005)	27.65
*Sparisoma cretense* (Milazzo)	I	1994 (1992–1995)	90.57
*Sphyraena viridensis*	I	1998 (1997–1999)	262.36
*Sprattus sprattus*	D	1995 (1994–1996)	150.59

The trend (increase ‘I’, decrease ‘D’ or fluctuate ‘F’), the year of statistically significant change (with CI at the 95% level) and results of the modified F test are showed for the species that contribute significantly in the SIMPER analysis. Confidence interval not shown because outside data time interval;

+ Increasing in last 10 years.

Probability of the Sup F <0.001 in all cases.

## Discussion

The six species that contributed the greatest to increased fish numbers over time (i.e. *Sphyraena viridensis*. *Caranx crysos*, *Sparisoma cretense*, *Coryphaena hippurus* and *Balistes capriscus*) are thermophilic fishes, typical from the southern sectors of the Mediterranean and their increase is consistent with what we would expect with climate warming. Remarkably the recent literature is rich in records of these species moving northward with respect to their previously known distribution. In particular, published observations are available for *S. viridensis* [33], [34], *C. crysos* [35], [36], *S. cretense* [37]–[39], *C. hyppurus* [40], *B. capriscus* [35], [41]. Moreover, for *C. crysos* [42], [43] and *B. capriscus* [44] northward expansions have been also documented in the Atlantic Ocean. Our data confirms that these organisms are good indicators of changes associated with warming in the marine environment [3], [12]. The group “DECREASE” (“D”) was represented by 42 species that are important for commercial fisheries. The most important families for this group were Scombridae and Clupeidae, two heavily fished groups all over the world. Obviously for “D” species, local negative impacts such as fishery, habitat degradation and pollution represent confounding effects to the search of global related variability. Disentangling the response to climate change from the effects of overfishing is particularly difficult for commercial species, nevertheless the decline observed for some boreal species such as *Sprattus sprattus* confirmed the existing concern on their resilience under a regime of climate warming [13]. To identify the species that may be in danger with the increase of temperature is a critical task [45] and traditional/local ecological knowledge can be considered valid source of such information, especially when scientific information is unavailable [25]–[27].

Finally in the group “FLUCTUATE” (“F”), two fishes were cited more often: *Fistularia commersonii* and *S. cretense*. The former is a non-native species that entered the Mediterranean in 2000 through the Suez Canal [46] spreading soon afterwards across the entire Mediterranean Sea [47]–[49]. Despite rapid geographic spread, the current status of these populations in the Central and in the Western Mediterranean is uncertain. Here, Local Ecological Knowledge provided a coherent indication of the instability of these populations which showed a rapid increase in 2003–2004 and a sharp decline soon afterwards. It is therefore possible that this species is not fully adapted to the new conditions of these Mediterranean sectors or that their dynamics are influenced by environmental fluctuations.

As far as *S. cretense* is concerned, in the 1970s this parrotfish was considered common in the Strait of Sicily [50] but absent from the northern Sicily [51]. Many respondents reported an increase of this species but others, the oldest ones with more than 30 year of experience reported a period of decline in the 70s and in the 80s followed by a clear trend of increase in the last ten years. Interestingly, the occurrence of important past fluctuations of the Mediterranean parrotfish is confirmed by the existence of a few historical observations of this species northwards with respect to its present distribution; for example, along the coast of France [52] and in the Central [53] and North Adriatic [54]. Thus, this species is considered one of clearest indicators of meridionalization because of its increasing abundance over the last two decades.

The analysis of historical trends of abundance revealed coherent species responses for the different study locations. The only exception was *Scomber japonicus* and probably more data is needed to know the real history of this species in the Mediterranean. As far as increasing species are concerned, the breakpoint analysis identified critical changes in the late 1990s and early 2000s. Actually, the first evidences of northward expansion of the range of warm-water come from the 1990s [37], [55]–[58] and it is probably in the last decade that this phenomenon has become more apparent. A positive trend of increase of water temperature and important changes in the water circulation of the Mediterranean Sea are visible since the 1980s [13], [57] and this might be at the basis of the geographical spread and success of thermophilic biota. The critical changes described by the structural analysis are indicative of the species as being fully established or even abundant in the ecosystem, while scientific records typically detect vagrant individuals or the early stages of colonization [14]. Despite the low number of interviews (only thirty-two), the perceived increase of thermophilic taxa was clear and coherent across the different Mediterranean sub-regions. Nevertheless in the future, additional surveys could be used to achieve a more precise reconstruction of historical trends, especially from earlier years that are typically difficult to sample. As usually happens in oral history surveys, the information we got was unequally distributed throughout the time. In fact, given the limited number of living people who have an early experience, pre-1970 data were based on only 5 interviews, compared to 21 respondents to the pre-1980 period and 26 respondents to the pre-1990.

Surveying Local Ecological Knowledge about changes in fish presence and abundance provided historical information that otherwise cannot be obtained. Perceived changes in species abundance can be clearly influenced by fishing methods and equipment (e.g. trawl, pelagic fishery, nets, lines and so on) and this influence could be better address with more data. Moreover, increases of some other termophilic species, such as *Thalassoma pavo* [59], could have passed unnoticed, simply because these species are not captured. Therefore it will be important in the future to broaden the number of people involved and to consider different kinds of users of natural resources such as scuba divers and long time local residents.

In local knowledge it is reasonable that not all the subjects and episodes are equally retained. In this, the capture of an “new fish” was certainly a special event that resulted to be easily remembered by the fishermen. This media property of species “never seen before” increase the potentialities of Local Ecological Knowledge as monitoring tools for these unusual occurrences that are typically difficult to monitor [14]. This possibility should be seriously taken into consideration due to our increasing need to approach large-scale patterns in the marine environment, such as species distribution shifts under climate change scenarios. Due to the preliminary nature of our results, this pilot study will hopefully serve in the future as a guide to carry out large scale investigation. In fact, more data over a broader spatial scale would allow a better definition of species temporal trends and to link these changes to environmental variables, especially along South-North gradients of the Mediterranean Sea, such as along the Italian peninsula or the Spanish coasts. Conversely, studying east-west gradients will be necessary in order to better understand the relevance of non-native species in recent changes in Mediterranean biodiversity.
